# Pediatric Influenza-Associated Encephalopathy and Acute Necrotizing Encephalopathy — United States, 2024–25 Influenza Season

**DOI:** 10.15585/mmwr.mm7436a1

**Published:** 2025-09-25

**Authors:** Amara Fazal, Elizabeth J. Harker, Varsha Neelam, Samantha M. Olson, Melissa A. Rolfes, Katie Reinhart, Krista Kniss, Aaron Frutos, Jerome Leonard, Carrie Reed, Vivien G. Dugan, Haytham Safi, Theresa M. Dulski, Adrianna Stanley-Downs, Aaliya Bhatti, Isaac Armistead, Suchitra Rao, Carola Torres-Diaz, Ashlin Thomas, Andy Weigel, Michael Patten, Mallory Sinner, Dawn Nims, Crystal Mattingly, Valerie Gosack, Amy Voris, Jaime Redkey, Felicia A. Scaggs Huang, Danielle DeCesaris, Carrie Tuggle, Kristina A. Betters, Julie Hand, Anna Krueger, Dina Z. Potter, Curi Kim, Rachel Park, Sue Hong, Hannah E. Edelman, Sue Kim, Justin Henderson, Melissa McMahon, Jeffrey Sanders, David A. Hunstad, Emma L. Doran, Khalil Harbi, Derek Julian, Hannah Ball, John Dreisig, Deepam Thomas, Justin Faybusovich, Yomei P. Shaw, Nancy Eisenberg, Richa Chaturvedi, Ashleigh Faulstich, Rachel E. Wester, Donna L. Gowie, Nicholas Fisher, Melissa Sutton, Sameh W. Boktor, Jonah M. Long, Patricia Marshall, Abby L. Berns, Lindsey McAda, Sarah Winders, Pamela Gomez Pinedo, Jade Murray, Ta’Kindra Westbrook, Anna Unutzer, Scott Lindquist, Thomas E. Haupt, Kaylyn Baum, Molly Wilson-Murphy, Carol Glaser, Kathleen Harriman, James W. Antoon, Keith P. Van Haren, Adrienne G. Randolph, Andrew Silverman, Annabelle de St. Maurice, Sascha Ellington, Timothy M. Uyeki, Shikha Garg, CDC Influenza-Associated Encephalopathy Collaborators, CDC Influenza-Associated Encephalopathy Collaborators, DeJuana Grant, Wes Stubblefield, Sarah Labuda, Cassandra Lautredou, Lori Simmons, Kalyani McCullough, Charsey Porse, Pam Daily Kirley, Amanda Feldpausch, Samantha McChesney, Sayna Patel, Matthew Donahue, Sheila Giovanni, Layne Mounsey, Bethany Hodge, Victoria M. Carroll, Joyce Cohen, Angela G. Fowler, Juliana A. Jacoboski, Carley Perez Kauffman, Jevon McFadden, Ruth Lynfield, John Bos, Jessica Goswitz, George Turabelidze, Erica Wilson, Brittany Yarnell, Katie Schultis, Justin Frederick, Helen Giambrone, Mario Lugo, Chad Wetzel, Angie Elliott, Ashley Johnson, Courtney Swick, Derek Bumgardner, Devi Dwarabandam, Cindy Beard, Devin Raman, Candyce Taylor, Arianna Tomasello, Danika Williams, Bridget J. Anderson, Adam Rowe, Rachel I. Paneth-Pollak, Alice Yeung, Laurie Billing, Mridula Gupta, Teegan Plackowski, M. Andraya Hendrick, Arilene Novak, Amanda Hartley, Alison Bridendolph, Melissa Plantenga, Elizabeth Hans, Varun Shetty, Jennifer Shuford, Whitney Tillman, Vivienne Heines, Yvonne Boire, Jeni Nybo, Tigran Avoundjian, BreeAnna Dell, Lawrence Lee, Alisha Yamanaka, Suresh B. Boppana, William L. Burton, Charlotte V. Hobbs, Susan C. Hutto, Yarlini Vipulanandan, Allison P. Williams, Sandra R. Arnold, Kate Shapiro, Zain Alamarat, Ana Del Valle Penella, Priya Edward, Deepti Nagesh, Felice Adler-Shohet, Megan Langille, Rachel Burri, Stephen Tomek, Mustafa Bakir, Cassandra Collins, Ryan Hurtado, Andrea C. Pardo, Ayelet Rosenthal, David Zhang, Kelly Howell, Kedar Tilak, Joel I. Howard, Laura Hegarty-Moore, Daniel Droutman, M. Cecilia Di Pentima, Neil Rellosa, Beth K. Thielen, Neel Shah, Susana Chavez-Bueno, Timothy D. Minniear, Peyton Thompson, Melissa M. Campbell, Shivani Devaguptapu, Ibukunoluwa C. Kalu, Davina Neal, Mame Anna Fall, Morgan Davidson, Meghan Gray, Eric Brownhill, Kevin A. Cassady, Ankita P. Desai, Andrea Scioscia, Tina L. Bair, Lori Handy, Evelyn S. Pangonis, Jennifer Wall Forrester, Coralee Del Valle Mojica, Aria Mooney, Fibi Attia, Justin Morris, Max Habicht, Jessica Wharton, James H. Conway

**Affiliations:** ^1^Influenza Division, National Center for Immunization and Respiratory Diseases, CDC; ^2^Epidemic Intelligence Service, CDC; ^3^Arkansas Department of Health; ^4^Career Epidemiology Field Officer Program, Office of Readiness and Response, CDC; ^5^California Department of Public Health; ^6^Colorado Department of Public Health and Environment; ^7^University of Colorado School of Medicine–Children’s Hospital of Colorado, Aurora, Colorado; ^8^Florida Department of Public Health; ^9^Iowa Department of Health and Human Services; ^10^Illinois Department of Public Health; ^11^OSF HealthCare Saint Francis Medical Center, Peoria, Illinois; ^12^Indiana Department of Health; ^13^Riley Hospital for Children at Indiana University Health, Indianapolis, Indiana; ^14^Infection Prevention & Control Program, Cincinnati Children’s Hospital Medical Center, Cincinnati, Ohio; ^15^Department of Pediatrics, University of Cincinnati College of Medicine, Cincinnati, Ohio; ^16^Kentucky Department of Public Health; ^17^Vanderbilt University Medical Center, Nashville, Tennessee; ^18^Louisiana Department of Health; ^19^Maine Center for Disease Control and Prevention; ^20^Maryland Department of Health, Baltimore, Maryland; ^21^The Johns Hopkins University School of Medicine, Baltimore, Maryland; ^22^Michigan Department of Health and Human Services; ^23^Minnesota Department of Health; ^24^Washington University School of Medicine, St. Louis, Missouri; ^25^North Carolina Department of Health and Human Services; ^26^Nebraska Department of Health and Human Services; ^27^New Hampshire Department of Health and Human Services; ^28^New Jersey Department of Health; ^29^New Mexico Department of Health; ^30^University of New Mexico, Albuquerque, New Mexico; ^31^Nevada Department of Health and Human Services; ^32^New York State Department of Health; ^33^Ohio Department of Health; ^34^Oregon Health Authority – Public Health Division, Portland, Oregon; ^35^Pennsylvania Department of Health; ^36^Rhode Island Department of Health; ^37^South Carolina Department of Public Health; ^38^Tennessee Department of Health; ^39^Utah Department of Health and Human Services; ^40^Virginia Department of Health; ^41^Washington State Department of Health, Tumwater, Washington; ^42^Wisconsin Department of Health Services; ^43^Wyoming Department of Health; ^44^Department of Neurology, Neuroimmunology Center, Boston’s Children’s Hospital, Boston, Massachusetts; ^45^Influenza Associated Encephalopathy Analytic Group, Atlanta, Georgia; ^46^Stanford University, Stanford, California; ^47^Department of Anesthesiology, Critical Care and Pain Medicine, Boston’s Children’s Hospital, Boston, Massachusetts; ^48^Los Angeles County Department of Health, Los Angeles, California.; Alabama Department of Public Health; Arkansas Department of Health; California Department of Public Health; California Emerging Infections Program; Georgia Department of Public Health; Iowa Department of Health and Human Services; Cook County Department of Public Health; Indiana Department of Health; Kentucky Department for Public Health; Massachusetts Department of Public Health; Michigan Department of Health and Human Services; Office of Readiness and Response, CDC; Minnesota Department of Health; Missouri Department of Health and Senior Services; North Carolina Department of Health and Human Services; Nebraska Department of Health and Human Services; Three Rivers Public Health Department; Douglas County Health Department; Lincoln-Lancaster County Health Department; Dakota County Public Health; Sarpy/Cass Health Department; South Heartland District Health Department; Nevada Department of Health and Human Services; Southern Nevada Health District; Northern Nevada Public Health; New York State Department of Health; New York City Department of Health and Mental Hygiene; Ohio Department of Health; Medina County Health Department; Oregon Health Authority – Public Health Division; Tennessee Department of Health; Texas Department of State Health Services; Austin Public Health; Vermont Department of Health; Tacoma–Pierce County Health Department; Public Health – Seattle & King County; University of Alabama at Birmingham; University of Tennessee Health Science Center and Le Bonheur Children’s Hospital; Le Bonheur Children’s Hospital; Arkansas Children’s Hospital; Children’s Hospital Los Angeles; Harbor-UCLA Medical Center; Atrium Health Beverly Knight Olsen Children’s Hospital; Children’s Hospital of Illinois, OSF HealthCare; Ann & Robert Lurie Children’s Hospital of Chicago, Northwestern University; Comer Children’s Hospital; Northwestern Medicine; Children’s Mercy Hospital; University of Kentucky; Baystate Medical Center, Baystate Health; Rhode Island Hospital, Brown University Health System; UMass Chan Medical School – Baystate; Nemours Children’s Hospital; University of Minnesota; Washington University in St. Louis; Children’s Mercey Kansas City; University of Tennessee Health Science Center; Division of Infectious Diseases, Department of Pediatrics, University of North Carolina at Chapel Hill; Duke University Hospital; HCA Healthcare, Inc.; UNC Hospitals; New York–Presbyterian Morgan Stanley Children’s Hospital; Jacobi Medical Center; Nationwide Children’s Hospital; University Hospital’s Rainbow Babies & Children’s Hospital; Akron Children’s Hospital; University of Cincinnati College of Medicine; Children’s Hospital of Philadelphia; St. Luke’s University Health Network; Penn State Health Milton S. Hershey Medical Center; Hasbro Children’s Hospital Brown University Health System; Prisma Health; University of Wisconsin–Madison, School of Medicine and Public Health

**Summary**

**What is already known about this topic?**

Influenza-associated encephalopathy (IAE) is a rare, severe neurologic complication of influenza.

**What is added by this report?**

During the high-severity 2024–25 influenza season, 109 U.S. pediatric IAE cases were identified; 55% of affected children were previously healthy. Thirty-seven IAE cases were subcategorized as acute necrotizing encephalopathy (ANE), a severe form of IAE characterized by rapid neurologic decline and a poor prognosis. Overall, 74% of IAE patients were admitted to an intensive care unit, and 19% died; 41% of ANE patients died. Only 16% of vaccine-eligible IAE patients had received the 2024–25 influenza vaccine.

**What are the implications for public health practice?**

All children are at risk for severe neurologic complications of influenza. Annual influenza vaccination is recommended for all children aged ≥6 months to prevent influenza and associated complications, potentially including IAE.

## Abstract

In January 2025, CDC received several reports of deaths among children aged <18 years with a severe form of influenza-associated encephalopathy (IAE) termed acute necrotizing encephalopathy (ANE). Because no national surveillance for IAE currently exists, CDC requested notification of U.S. pediatric IAE cases from clinicians and health departments during the 2024–25 influenza season, a high-severity season with a record number of pediatric influenza-associated deaths. Among 192 reports of suspected IAE submitted to CDC, 109 (57%) were categorized as IAE, 37 (34%) of which were subcategorized as ANE, and 72 (66%) as other IAE; 82 reports did not meet IAE criteria and were categorized as other influenza-associated neurologic disease. The median age of children with IAE was 5 years and 55% were previously healthy, 74% were admitted to an intensive care unit, and 19% died; 41% of children with ANE died. Only 16% of children with IAE who were vaccination-eligible had received the 2024–25 influenza vaccine. Health care providers should consider IAE in children with encephalopathy or altered level of consciousness and a recent or current febrile illness when influenza viruses are circulating. Annual influenza vaccination is recommended for all children aged ≥6 months to prevent influenza and associated complications, potentially including severe neurologic disease such as IAE and ANE.

## Introduction

The 2024–25 influenza season was historicallysevere with the highest number of pediatricinfluenza-associateddeaths reported during a seasonal influenza epidemic since U.S. surveillance for these deaths began in 2004 (excluding the 2009–10 influenza A(H1N1)pdm09 pandemic). No U.S. surveillance for neurologic complications of influenza exists. Influenza-associated encephalopathy (IAE), a recognized complication of influenza, refers to neurologic syndromes triggered by influenza virus infection of the respiratory tract, resulting in a dysregulated host inflammatory response and leading to varying degrees of brain dysfunction (*1*,*2*). One of the most severe forms of IAE is acute necrotizing encephalopathy (ANE), a condition that disproportionately affects children and is characterized by rapid neurologic decline and neuroimaging with evidence of necrosis or hemorrhage involving the thalami; ANE has a poor prognosis and can result in lasting neurologic sequelae or death (*2*,*3*).

In January 2025, CDC was alerted to several deaths of children with influenza-associated ANE (*4*). In response, CDC requested notification from clinicians and health departments of possible cases of pediatric IAE, including influenza-associated ANE, to better characterize these syndromes in the U.S. during the 2024–25 influenza season. This report describes cases reported in response to CDC’s request.

## Methods

### Data Collection

On February 28, 2025, CDC released a call for cases of IAE in persons aged <18 years via the EpidemicInformationExchange|Epi-X, asking clinicians and health departments to contact CDC if cases fulfilled CDC’s IAE surveillance criteria (Box) (*4*). Case report forms^†^ were completed by clinicians, public health practitioners, and partners from CDC-sponsored surveillance networks (i.e., FluSurv-NET|FluView, NewVaccineSurveillanceNetwork|NVSN, and Influenza-AssociatedPediatricMortality|CDC) if surveillance criteria were met and electronic health record (EHR) data were available.

BOX **Required surveillance criteria for pediatric influenza-associated encephalopathy investigation — United States, 2024–25 influenza season**

1. Patient age <18 years

2. Admitted to a U.S. acute care hospital or pronounced dead in a U.S. emergency department during October 1, 2024–May 30, 2025

3. Laboratory-confirmed influenza virus infection within 14 days preceding hospital evaluation, during hospitalization, or in respiratory specimens collected postmortem

4. Documented neurologic abnormalities (meets one or more of the following criteria):

- Diagnosis of encephalopathy or encephalitis
- Neurologic signs or symptoms, including but not limited to
  - seizures
  - altered mental status
  - delirium
  - decreased level of consciousness
  - lethargy
  - hallucinations
  - personality changes lasting >24 hours
- Neuroimaging abnormalities such as brain edema, brain inflammation, or brain lesions
- Electroencephalogram abnormalities (unspecified)
- Abnormal brain autopsy findings, if available, for children who died

### Case Categorization

Neuroimaging findings and discharge diagnoses underwent review by a physician to categorize cases as IAE or influenza-associated neurologic disease. IAE cases were subcategorized into ANE (those with compatible neuroimaging findings or an ANE discharge diagnosis) or other IAE. ANE cases were defined as probable if neuroimaging reports described bilateral thalamic inflammatory lesions and possible if the patient received a discharge diagnosis^§^ of ANE without these neuroimaging findings. IAE cases that did not fulfill ANE criteria were categorized as other IAE if a discharge diagnosis of IAE was reported. All other submitted cases were categorized as influenza-associated neurologic disease and are described separately (SupplementaryTable). Reports were excluded if co-detection of a neuroinvasive pathogen in addition to influenza was reported.

Demographics and clinical characteristics and outcomes were described overall and by case categorization. Deidentified data were collected and stored in a REDCap database (version 15.5.8; Vanderbilt University) hosted at CDC, and SAS software (version 9.4; SAS Institute) was used for all analyses. Missing responses were excluded from denominators. This activity was reviewed by CDC, deemed not research, and was conducted consistent with applicable federal law and CDC policy.^¶^

## Results

CDC received 192 reports that met surveillance criteria (Figure). Among those, 109 cases were categorized as IAE, 37 (34%) of which were subcategorized as ANE (Table). An additional 82 reports were categorized as influenza-associated neurologic disease, a category for those cases that did not meet the IAE case definition; demographics and clinical characteristics, influenza antiviral treatment, and illness severity of these cases were generally similar to those of IAE cases and are described separately (SupplementaryTable). Percentages of characteristics were calculated among those patients with available information.

**FIGURE:**
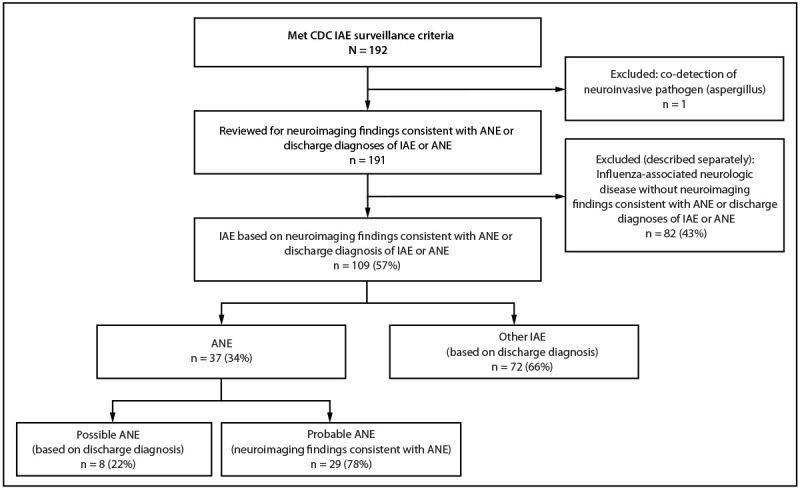
Categorization of cases of pediatric influenza-associated encephalopathy reported to CDC — United States, 2024–25 influenza season **Abbreviations:** ANE = acute necrotizing encephalopathy; IAE = influenza-associated encephalopathy.

**TABLE:** Characteristics of reported pediatric influenza-associated encephalopathy cases — United States, 2024–25 influenza season

Characteristic	All cases		ANE		Other IAE	
	n/N*	Column %	n/N	Column %	n/N	Column %
**Total (row %)**	**109**	**100**	**37**	**34**	**72**	**66**
Median age, yrs (IQR)	**5 (3–10)**	**—**	4 (1–7)	—	6 (4–10)	—
**Age group, yrs**						
0–4	**44/109**	**40**	22/37	59	22/72	31
5–11	**46/109**	**42**	11/37	30	35/72	49
12–17	**19/109**	**17**	4/37	11	15/72	21
**Female sex**	**49/107**	**46**	18/37	49	31/70	44
**Race and ethnicity^†^**						
Asian, non-Hispanic	**7/102**	**7**	4/35	11	3/67	4
Black or African American, non-Hispanic	**19/102**	**19**	4/35	11	15/67	22
Hispanic or Latino	**16/102**	**16**	8/35	23	8/67	12
White, non-Hispanic	**53/102**	**52**	18/35	51	35/67	52
Other, non-Hispanic	**7/102**	**7**	1/35	3	6/67	9
**U.S. Census Bureau region^§^**						
Northeast	**31/109**	**28**	6/37	16	25/72	35
Midwest	**26/109**	**24**	8/37	22	18/72	25
South	**31/109**	**28**	14/37	38	17/72	24
West	**21/109**	**19**	9/37	24	12/72	17
**Hospital admission month^¶^**						
Before influenza peak (Oct–Dec)	**13/109**	**12**	5/37	13	8/72	11
During influenza peak (Jan–Feb)	**71/109**	**65**	29/37	78	42/72	58
After influenza peak (Mar–May)	**25/109**	**23**	3/37	8	22/72	31
Underlying medical conditions**^,††^						
None	**58/106**	**55**	18/35	51	40/71	56
At least one	**48/106**	**45**	17/35	49	31/71	44
Asthma	**12/106**	**11**	3/35	9	9/71	13
Seizure disorder	**10/106**	**9**	5/35	14	5/71	7
Neurologic or neuromuscular disease	**15/106**	**14**	5/35	14	10/71	14
**Signs and symptoms on admission^§§^**						
Altered mental status^¶¶^	**93/106**	**88**	32/35	91	61/71	86
Fever	**92/108**	**85**	34/37	92	58/71	82
Headache	**22/86**	**26**	5/28	18	17/58	29
Respiratory tract symptoms	**91/104**	**87**	33/36	92	58/68	85
Seizures	**56/94**	**60**	28/32	87	28/62	45
Illness onset to neurologic symptom onset days, (IQR)***	**2 (1–3)**	**—**	2 (1–3)	—	2 (1–4)	—
**Influenza vaccine status** ^†††^						
Received the 2024–25 seasonal influenza vaccine ≥14 days before illness onset	**15/93**	**16**	4/30	13	11/63	17
**Influenza antiviral treatment**						
Received an influenza antiviral^§§§^	**86/102**	**84**	31/33	94	55/69	80
Illness onset to antiviral start date, days (IQR)***	**3 (1–4)**	**—**	2 (2–4)	—	3 (2–4)	—
Started before admission	**8/80**	**10**	2/27	7	6/53	11
Started on or after admission	**72/80**	**90**	25/27	93	47/63	89
**Other treatment**						
Immunomodulators***	**17/80**	**21**	14/25	56	3/55	5
Intravenous immunoglobulin***	**23/79**	**29**	16/24	67	7/55	13
Plasma exchange***	**15/80**	**19**	11/25	44	4/55	7
Systemic corticosteroids	**52/98**	**53**	29/33	88	23/65	35
Vasopressors***	**25/79**	**32**	17/24	71	8/55	15
**Influenza virus type or subtype**						
Influenza A	**97/109**	**89**	34/37	92	63/72	87
Influenza A (H1N1)	**37/59**	**63**	13/23	56	24/36	67
Influenza A (H3N2)	**22/59**	**37**	10/23	43	12/36	33
Influenza B	**12/109**	**11**	3/37	8	9/72	12
**Bacterial, viral, or fungal detection^¶¶¶^**	**13/109**	**12**	5/37	13	8/72	11
Neuroimaging performed****						
Yes	**102/108**	**94**	37/37	100	65/71	92
No	**6/108**	**6**	0	0	6/71	8
Abnormal findings^††††^	**68/102**	**67**	36/37	97	32/65	49
**Illness severity**						
Median length of hospitalization among survivors, days (IQR)^§§§§^	**9 (3–24)**	**—**	30 (18–38)	—	6 (3–17)	—
Median length of hospitalization among patients who died, days (IQR)^§§§§^	**4 (3–7)**	**—**	4 (3–7)	—	5 (1–8)	—
Pneumonia diagnosis at admission	**19/101**	**19**	6/34	18	13/67	19
Admitted to an ICU	**80/108**	**74**	37/37	100	43/71	61
Invasive mechanical ventilation	**59/109**	**54**	33/37	89	26/72	36
Not at neurologic baseline at discharge^¶¶¶¶^	**33/70**	**47**	12/13	92	21/57	37
Death	**21/109**	**19**	15/37	41	6/72	8

**Abbreviations:** ANE = acute necrotizing encephalopathy; IAE = influenza-associated encephalopathy; ICU = intensive care unit.

* Denominators are adjusted throughout the table to exclude missing and unknown responses.

^†^ Children with multiple races selected and non-Hispanic ethnicity selected were categorized as “Other, non-Hispanic.”

^§^ Based on state of residence. Censusregionsanddivisions|U.S.CensusBureau

^¶^ Peak based on national influenza activity for the 2024–25 influenza season. WeeklyUSInfluenzaSurveillanceReport:KeyUpdatesforWeek35,endingAugust30,2025|FluView|CDC

** Underlying medical conditions include the following categories: developmental (e.g., autism and attention deficit hyperactivity disorder), prematurity for those aged <2 years, immunocompromising conditions, chronic metabolic disease, genetic or inborn errors of metabolism, blood disorders, lung disease, cardiovascular disease, renal disease, gastrointestinal disease, rheumatologic disease, and obesity.

^††^ Two children had underlying medical conditions that can predispose to encephalopathy in the setting of a systemic stressor such as influenza virus. These conditions include an inborn error of metabolism (one) and a leukodystrophy (one).

^§§^ Numbers are not mutually exclusive.

^¶¶^ Altered mental status includes delirium, personality changes, hallucinations, and decreased level of consciousness.

*** Optional survey questions included illness onset date, neurologic symptom onset date, and use of other treatments.

^†††^ Among those aged ≥6 months and thus eligible for influenza vaccination. Admission date was used for five IAE patients for whom the illness onset date was not available.

^§§§^ Seventy-two patients received oseltamivir alone, one received oseltamivir and baloxavir marboxil, six received oseltamivir and peramivir, six received peramivir alone, and one was missing influenza antiviral type information.

^¶¶¶^ Co-detections were reported from any time during hospitalization for any of the following specimen sources: blood, urine, respiratory tract, peritoneal fluid, or cerebrospinal fluid.

**** Neuroimaging performed included computed tomography of the head and magnetic resonance imaging of the brain.

^††††^ Percentage of patients with neuroimaging performed.

^§§§§^ To discharge (for survivors) or death; data were missing for five IAE patients (two ANE and three other IAE).

^¶¶¶¶^ Among patients who survived, were no longer hospitalized, and for whom survey data were available.

### Characteristics of All Patients with Influenza-Associated Encephalopathy

Among the 109 IAE cases with available data, median patient age was 5 years (IQR = 3–10 years) (Table). Approximately one half of patients were female (46%) and non-Hispanic White (52%). Overall, 97 (89%) patients had influenza A virus infection; among the 59 (61%) cases with influenza A virus subtype available, 37 (63%) had A(H1N1)pdm09 and 22 (37%) had A(H3N2). Approximately one half (55%) of patients were previously healthy with no underlying medical conditions.** Signs and symptoms most commonly reported at initial assessment were altered mental status (88%), respiratory symptoms (87%), and fever (85%). Among patients with ANE, 87% had seizures at the time of admission; among the other IAE patients, seizures were noted in 45% of cases.

Neurologic symptoms commenced a median 2 days after illness onset^††^ (IQR = 1–3 days). Overall, neuroimaging was received by 94% of IAE patients; abnormal findings were reported for 97% of ANE patients and 49% of other IAE patients. Influenza antiviral treatment was administered to 84% of IAE patients, beginning a median of 3 days after illness onset, and among 90% of all IAE patients, antiviral treatment started on or after the date of hospital admission. Among all IAE patients, 74% were admitted to an intensive care unit (ICU), 54% received invasive mechanical ventilation, and 19% died. Among the 70 survivors with information on neurologic status at discharge, 47% had not returned to their neurologic baseline.^§§^ Among 93 patients with information on seasonal influenza vaccination, 15 (16%) had received ≥1 dose of the 2024–25 seasonal influenza vaccine ≥14 days before illness onset.^¶¶^

### Characteristics of Patients with Acute Necrotizing Encephalopathy

Among the 37 IAE cases subcategorized as ANE with available data, the median patient age was 4 years (IQR = 1–7 years). Approximately one half (51%) of patients were previously healthy. Four (13%) of 30 ANE patients had received ≥1 dose of the 2024–25 seasonal influenza vaccine ≥14 days before illness onset. Among patients with data available on interventions provided, influenza antivirals were received by 94%, systemic corticosteroids by 88%, intravenous immunoglobulin by 67%, other immunomodulators (e.g., tocilizumab, baricitinib, or anakinra) by 56%, and plasma exchange by 44%. All patients with ANE were admitted to an ICU, and 89% received invasive mechanical ventilation. Fifteen (41%) patients with ANE died. Among 13 survivors with information about neurologic sequelae at discharge, only one had returned to neurologic baseline. The median hospital stay was 16 days (IQR = 4–31 days) for all ANE patients and 30 days (IQR = 18–38 days) among survivors. ANE patients who died were hospitalized for a median of 4 days (IQR = 3–7 days) before death.

## Discussion

During the 2024–25 influenza season, 109 cases of IAE in children were reported to CDC; approximately one third of these children (37; 34%) had ANE. These patients comprise the largest case series of children with IAE in the United States reported to date. Most children with IAE had fever and altered mental status at the time of hospital evaluation, and neurologic symptoms began shortly after influenza symptom onset. Many children experienced critical illness: 74% were admitted to an ICU, and 54% received invasive mechanical ventilation. Approximately one half of these children were previously healthy with no underlying medical conditions.

Although many children with IAE had neuroimaging abnormalities reported, neuroimaging abnormalities might or might not be present in patients with IAE (*2*,*5*). Influenza virus type and influenza A virus subtype distribution in these cases were generally consistent with national circulation of seasonal influenza viruses.

Patients reported to CDC with ANE had more severe illness than did those with other IAE; ANE patients had high mortality (41%) and rapid progression to death, and all patients had critical illness. Hospital length of stay was prolonged among survivors, and only one survivor had returned to neurologic baseline at discharge. Patients with ANE had seizures at hospital evaluation almost twice as often (87%) as did patients with other IAE (45%). Overall, only 13% of patients with ANE reported to CDC had received influenza vaccination during the 2024–25 season. 

A recently published U.S. clinical case series described influenza-associated ANE among 41 children during the 2023–24 and 2024–25 influenza seasons and observed that only 16% of patients had received seasonal influenza vaccination among 38 with known vaccination status, 76% had no significant medical history, and 27% died within days of symptom onset (*6*). ANE cases during the 2024–25 influenza season might have been reported to both this investigation and the 2023–25 case series, but the studies differed in methodology (including level of clinical detail collected and reviewed, case recruitment strategies, and exclusion criteria). Overlap among the 37 IAE cases subcategorized as ANE reported in this public health investigation and the 41 reported in that case series cannot be quantified.

Since 2010, CDC and the Advisory Committee on Immunization Practices have recommended annual influenza vaccination for all persons aged ≥6 months (*7*). Influenza vaccination can prevent influenza illness and reduce the severity of influenza in children who do become ill, including reduction in occurrence of critical and life-threatening influenza (CDC|BenefitsoftheFluVaccine) (*8*). Influenza vaccination has also been found to reduce influenza-associated hospitalization and emergency department visits in children (*9*). Despite these known benefits, pediatric influenzavaccinationcoverage has declined in recent years*** and only 16% of vaccine-eligible IAE patients reported to CDC had received the 2024–25 influenza vaccine.

Preadmission oseltamivir treatment among IAE patients was low. Outpatients with suspected or confirmed influenza who are at high risk for influenza complications are recommended to start influenza antiviral treatment as soon as possible after symptom onset; antiviral treatment might also be considered for patients who are not at higher risk (CDC|AntiviralMedications). Whether influenza antiviral therapy affects the development or progression of IAE is unknown; however, one study demonstrated that oseltamivir treatment of influenza in outpatients aged 5–17 years was associated with a reduced risk for hospitalization with serious neuropsychiatric events, including neurologic events such as seizure, altered mental status, and encephalitis (*10*).

### Limitations

The findings in this report are subject to at least three limitations. First, included cases are a convenience sample and might not be representative of all U.S. IAE cases during the 2024–25 influenza season. Second, categorization of IAE cases relied partially on discharge diagnoses, which likely underrepresent the true incidence of IAE, as IAE has no consensus standardized diagnostic criteria and might be underdiagnosed. Finally, deidentified data available for analysis were based on data abstracted from EHRs and reported on the surveillance case report form. Therefore, reported data did not necessarily include the complete clinical course and all clinical or laboratory data, neuroimaging reports, or primary neuroradiographic images.

### Implications for Public Health Practice

IAE is a serious neurologic complication of influenza that can affect healthy children as well as those with underlying medical conditions. During influenza season, parents and caregivers of children with neurologic signs and symptoms (e.g., seizures, hallucinations, or altered level of consciousness) in conjunction with fever or respiratory symptoms should seek care urgently. Health care providers should consider IAE in children with recent or current febrile illness with encephalopathy, monitor these children for clinical deterioration, and initiate appropriate supportive care. 

Annual influenza vaccination is recommended for all children aged ≥6 months to prevent influenza and associated complications, potentially including neurologic disease such as IAE and ANE. Early influenza antiviral treatment is recommended as soon as possible for all children with influenza who are hospitalized or at increased risk for influenza complications because of age or presence of comorbidities.

No consensus standardized diagnostic or surveillance case definitions for IAE currently exist. Additional measures are needed to develop and implement surveillance to improve understanding of the incidence, potential risk factors, severity, and public health impact of IAE in the United States. 

CDC is integrating surveillance for IAE and ANE into existing CDC-sponsored surveillance systems for the 2025–26 influenza season to better understand these serious and potentially preventable complications of influenza.

## Footnotes

† Case report form questions covered demographics, influenza testing and vaccination status, symptoms on admission, clinical course details available in EHR, and discharge diagnoses. Optional data elements, such as illness onset date and findings and impressions from neuroimaging reports, were included on the case report form and described among cases with available data.

§ Patients who died had discharge diagnoses selected on the case report form; postmortem diagnoses reported were likely derived from the death report in the EHR, although other sources, including death certificates, might have been reviewed to complete that section of the case report form.

¶ 45 C.F.R. part 46.102(l)(2), 21 C.F.R. part 56; 42 U.S.C. Sect. 241(d); 5 U.S.C. Sect. 552a; 44 U.S.C. Sect. 3501 et seq.

** Among all patients with IAE, 58% (63 of 109) were considered to be at increased risk for complications of influenza based on age <2 years or age ≥2 years with underlying medical conditions. PeopleatIncreasedRiskforFluComplications|Influenza(Flu)|CDC

†† Among patients with both a neurologic symptom onset date and an illness onset date available.

§§ Among patients who survived, were no longer hospitalized, and for whom survey data were available.

¶¶ Among patients aged ≥6 months who were eligible for influenza vaccination. Admission date was used for one ANE patient and four other IAE patients with no illness onset date available.

*** Data for the 2024–25 influenza season as of April 26, 2025.
